# Metastatic Non-Clear Cell Renal Cell Carcinoma: An Evidence Based Review of Current Treatment Strategies

**DOI:** 10.3389/fonc.2015.00067

**Published:** 2015-04-08

**Authors:** Alexander Sankin, A. Ari Hakimi, James J. Hsieh, Ana M. Molina

**Affiliations:** ^1^Memorial Sloan-Kettering Cancer Center, New York, NY, USA; ^2^Weill Cornell Medical College, New York, NY, USA

**Keywords:** non-clear cell, renal cell carcinoma, targeted agents, treatment, metastatic

## Abstract

Much progress has been made in the treatment of metastatic renal cell carcinoma (RCC) over the last decade, with the development of agents that block the vascular endothelial growth factor (VEGF) pathway or the mammalian target of rapamycin (mTOR) pathway. The incorporation of these agents into treatment algorithms has been the result of carefully conducted clinical trials leading to Food and Drug Administration (FDA) approval and subsequent adoption as the current standard of care. These trials, however, were dominated by patients with clear cell renal cell carcinoma (ccRCC), and little data are currently available on the treatment of non-clear cell renal cell carcinoma (nccRCC). nccRCC encompasses a biologically heterogeneous group of kidney tumors that portend very diverse prognoses and responses to therapy. This review is a pathway based approach that highlights the current systemic treatment strategies for metastatic nccRCC.

## Introduction

### Epidemiology of nccRCC

Kidney cancer is the eighth most common malignancy in the United States. There are approximately 65,000 new cases and 13,000 deaths per year due to kidney cancer (1), making it the most lethal of the common urologic malignancies. RCC is the predominant form of primary renal malignancies. RCC is actually a family of tumors, each with distinct genetic landscapes resulting in a heterogeneous group of disease processes. The neoplasms categorized as RCC’s exhibit diverse growth patterns, metastatic potentials, and responses to treatment (2). By far the most common RCC is clear cell [clear cell renal cell carcinoma (ccRCC)], which accounts for 75–80% of all primary kidney malignancies (3). The next most prevalent histologies include papillary (10–15%), chromophobe (5%), collecting duct/medullary carcinomas (1–2%), translocation associated RCC (<1%), and unclassified (~5%).

### Subtypes of nccRCC

Papillary RCC’s are tumors characterized histologically by a predominantly papillary cellular architecture (4). These tumors arise from the proximal convoluted tubule portion of the nephron (5). Papillary tumors are further subdivided into type 1 and type 2 classifications. Sporadic type 1 papillary RCC’s typically present as multifocal tumors, although they tend to exhibit slow growth rates and low metastatic potential when compared to ccRCC (6). It has been suggested that this malignancy may occur at a higher rate in black patients (7). These tumors are closely linked to genetic alterations in the met oncogene (c-Met), which encodes the receptor for hepatic growth factor (HGF) (8). A germline mutation in *c-Met* is the etiology for hereditary papillary renal carcinoma, a familial syndrome that causes bilateral, multifocal papillary type 1 tumors in massive quantities (sometimes greater than 1,000 tumors per kidney) (9). Papillary type 1 malignancies can also arise in a sporadic fashion. These tumors are commonly associated with *c-Met* gene amplification and, less often, mutations within the *c-Met* gene.

In contrast, type 2 papillary RCC tends to have a more aggressive clinical course leading to higher rates of metastasis and decreased survival (10). The inherited form of the disease has been associated with germline mutations in the fumarate hydratase gene (FH) (11). This gene is intimately involved in the Krebs cycle and when inactivated leads to an accumulation of fumarate and stabilization of the HIF1-α complex (12). Patients with this condition, also known as hereditary leiomyomatous RCC, present with cutaneous and uterine leiomyomas as well as kidney cancer (13). The renal tumors should not be managed expectantly in those with this condition as they tend to behave aggressively (14). They are typically locally infiltrative into the surrounding normal parenchyma and should be resected with a wide margin to prevent local recurrence (15).

Chromophobe RCC accounts for 5% of all primary renal malignancies. These tumors arise from the intercalated cells of the distal nephron and appear histologically as solid sheets of cells with eosinophilic cytoplasm (5). These tumors typically behave indolently and rarely metastasize (16). Mutations in the folliculin gene located on chromosome 17 lead to Birt Hogg Dubé disease, characterized by cutaneous fibrofolliculomas, pneumothoraces, and chromophobe RCC (17). Patients with this condition may also present with oncocytomas and hybrid oncocytic renal masses.

One of the least common and highly aggressive forms of RCC is the collecting duct subtype. These tumors account for roughly 1–2% of all primary renal tumors. They arise from the collecting duct epithelia and are histologically and genetically closely linked to urothelial tumors of the upper tract (5). They are more likely to present as advanced disease when compared to other renal tumors (18). A variant of this tumor known as medullary carcinoma presents most commonly in patients with sickle cell trait (19) and is associated with the loss of SMARCB1/INI1 expression (20). Overall, collecting duct carcinomas metastasize early and have a poor prognosis. Additionally, there are very few effective systemic treatments available for this disease.

There are many chromosomal translocations causing disruption in the microphthalmia-associated transcription factor (MiT) family of genes that have been implicated in a subtype of RCC known as translocation associated RCCs (21). The three main culprit genes are Transcription factor E3 (TFE3), Transcription factor EB (TFEB), and MITF (14). These tumors have a propensity to present at a young age and represent up to 45% of kidney tumors in children (22). The recommended treatment of translocation associated RCCs is immediate surgical resection along with lymph node dissection, as they can often present with early nodal metastasis.

Another rare subtype of RCC is unclassified RCC (~5%). This family of tumors typically contains unfavorable histology and result in poor clinical outcomes, although it is possible that this is a consequence of these tumors presenting with advanced clinicopathologic features compared to ccRCC (23).

### Genetic signatures in RCC

Great strides in our understanding of the genomic landscape of RCC have been made in the last decade. This last year marked the completion of two large scale studies that comprehensively analyzed the somatic alterations responsible for ccRCC (24, 25). These multiplatform genetic analyses of over 500 tumors characterized the oncogenic signature of this disease. While this effort sheds some light on driver genes of RCC, the study was limited to the clear cell subtype. The altered pathways identified in these analyses may not play the same role in the tumorigenesis of non-clear cell renal cell carcinoma (nccRCC). For example, ccRCC is a disease with a strong association with 3p loss, an alteration that is not typically present in nccRCC. More recently, the Cancer Genome Atlas (TCGA) sequenced 66 primary chromophobe RCC tumors, revealing an overall simpler genomic landscape than that of its clear cell counterpart (26). Interestingly, 23% of chromophobe tumors contained at least one mutation in the mTOR signaling pathway (mTOR, NRAS, TSC1/2), indicating that therapeutic targeting of this pathway may be a worthwhile strategy (27).

### Clinical outcomes in metastatic nccRCC

**Figure 1:**
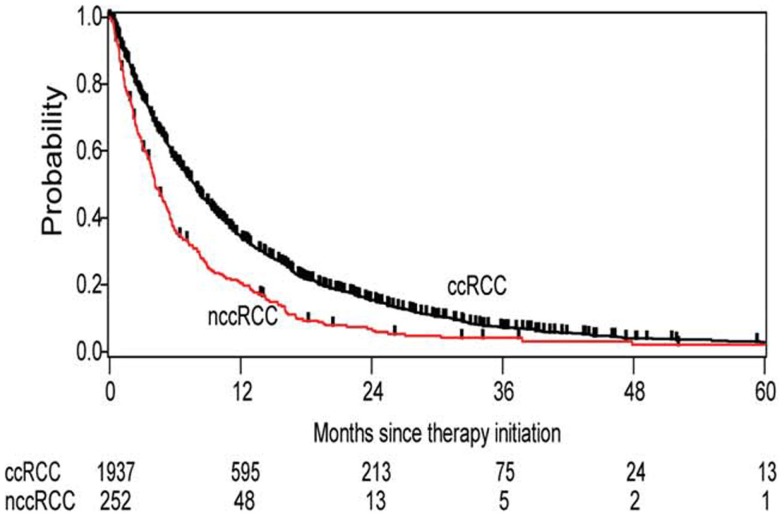
**Kaplan–Meier estimates of overall survival in all patients with clear cell histology vs non-clear cell histology**.

Data from the International Metastatic Renal Cell Carcinoma Database Consortium (IMDC) show that the diversity of renal tumors is not just limited to their biologic blueprints, but is reflected in its behavior, which impacts clinical outcomes as well (28). nccRCC patients had a significantly worse overall survival than their clear cell counterparts (12.8 vs 22.3 months, respectively) (Figure 1). The nccRCC patients were then subdivided into specific histologies, revealing the best survival among patients with chromophobe tumors, followed by papillary tumors, and then unclassified.

## Treatment Options

The systemic treatment of metastatic RCC has been largely dictated by clinical trials in which clear cell histologies predominate the study population. It would be inaccurate to extrapolate these results to tumors of non-clear cell histology due to the diversity of genomic landscape and molecular architecture from which they arise (29). Due to the scarcity of level 1 data on these tumors, there is no current consensus on appropriate first line treatment. The National Comprehensive Cancer Network (NCCN) recommends enrollment in a clinical trial as the preferred treatment option for a patient presenting with metastatic non-clear cell disease (30). Other therapeutic options include temsirolimus, sorafenib, sunitinib, pazopanib, axitinib, everolimus, bevacizumab, or erlotinib. Of these treatments, only temsirolimus and bevacizumab have been studied in a phase 3 clinical trial in which the effect of various tumor histologies was independently analyzed. The following section highlights the cellular pathways presumed to be altered in non-clear cell tumors and the targeted agents that may yield a clinical response.

## NCCN Recommended Agents

### mTOR pathway

Mammalian target of rapamycin (mTOR) is a serine–threonine kinase that serves as a central regulator of cell growth and proliferation, metabolism, and angiogenesis (31). mTOR is a downstream component of the PI3K/AKT pathway, which is activated by the tyrosine kinase cell surface receptors of insulin-like growth factor (IGF), epidermal growth factor (EGF), platelet-derived growth factor (PDGF), and vascular endothelial growth factor (VEGF). The dysregulation of the PI3K/AKT/mTOR pathway is known to be associated with many human cancers, including RCC.

**Figure 2:**
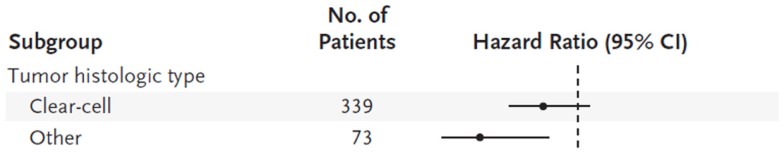
**Hazard ratios (indicated by circles) with 95% confidence intervals (indicated by horizontal lines) are shown for subgroups of patients receiving interferon-α or temsirolimus**.

Temsirolimus, an mTOR inhibitor, was approved for treatment of advanced RCC after the ARCC trial. This large, multi-institutional phase 3 trial studied the effects of temsirolimus as first line therapy for poor-prognosis advanced RCC (32). A total of 626 patients were randomly assigned to temsirolimus, interferon-α, or combination therapy. Patients randomized to the temsirolimus group had a longer overall survival compared to the interferon and combination group (10.9 vs 7.3 and 8.4 months, respectively). While this study included patients with all histologies of RCC, the data suggested a more pronounced survival advantage in patients with non-clear cell histology, which prompted a subgroup analysis (Figure 2). In this analysis, they identified 73 patients with nccRCC (37 randomized to temsirolimus arm and 36 to the interferon-α arm) (33). Within this cohort, they observed an improved clinical benefit (defined as complete or partial response or stable disease ≥24 weeks) of temsirolimus over interferon alpha (35.1 vs 8.3%, respectively). They also observed a greater degree of improvement among the non-clear cell subgroup compared to the clear cell patients, but this finding was not statistically significant. The authors speculated that the clinical improvement observed with temsirolimus on non-clear cell tumors may be due to the critical role of angiogenesis in all RCC histologies as well as the close relationship between mTOR and *c-Met* pathways. Temsirolimus is the only agent with a NCCN category 1 recommendation (in poor prognosis patients according to MSKCC criteria) for metastatic non-clear cell disease (30). It has a category 2A recommendation for all other prognostic risk groups.

Everolimus, another mTOR inhibitor, has been recently evaluated in two phase 2 trials in patients with metastatic nccRCC. The RAPTOR trial (RAD001 in Advanced Papillary Tumor Program in Europe) conducted by Escudier et al. enrolled patients with metastatic papillary RCC and no prior therapy to receive first line everolimus (34). Of the 92 patients studied, 59% of them had stable disease after 6 months of therapy. Progression free survival was 7.8 months and at least half of the patients were alive at 20 months. Another trial by Koh et al. enrolled 43 patients with metastatic non-clear cell disease to be treated with everolimus, 23 of whom had received prior VEGF therapy (35). Progression free survival was 5.2 months and partial response was observed in five patients (10%). Subgroup analysis revealed a substantially longer progression free survival of 13.1 months in patients with chromophobe tumors (*n* = 8).

### VEGF pathway

Angiogenesis is a crucial component of tumor growth and invasion (36). This process is in part driven by migration and proliferation of endothelial cells via activation of the VEGF receptor (a tyrosine kinase cell surface receptor). Dysregulated cancer cells secrete abnormally high levels of angiogenic factors including VEGF in order to recruit nearby vascular endothelial cells, which in turn proliferate and form new blood vessels to deliver oxygen to the highly metabolic tumors.

Two recent trials have compared sunitinib and everolimus as first line therapy in metastatic nccRCC. The ESPN trial randomized patients with non-clear cell disease to receive either sunitinib or everolimus as first line therapy (37). Interim analysis of 67 patients suggested an overall and progression free survival advantage in the sunitinib arm (16.2 vs 14.9 and 6.1 vs 4.1 months, respectively). Additionally, a *post hoc* sub-group analysis of metastatic nccRCC patients (*n* = 66) enrolled in the RECORD-3 trial suggested a progression free survival advantage of sunitinib (7.2 vs 5.1 months) (38).

There have been many single arm phase 2 trials studying the role of VEGF inhibitors in patients with advanced nccRCC. Results have been variable and overall treatment effect appears to be poor to fair. A recent phase 2 trial studied the effect of sunitinib for treatment of metastatic nccRCC (39). In this study, 23 patients with various non-clear cell tumor histologies were enrolled (35% of patients had papillary RCC). Progression free survival was 5.5 months for all patients (similar to the 5.6 months observed in the papillary RCC subgroup). Overall response rate to sunitinib was poor, with only one patient achieving a partial response (of note, the patient had unclassified histology). Another phase 2 trial treated 31 patients with non-clear cell tumors of various histologies with sunitinib (40). Most patients (71%) had papillary RCC. Overall response rate was 36% and median progression free survival was 6.4 months. In the largest phase 2 trial to date studying the role of sunitinib in advanced non-clear cell RCC, Tannir et al. prospectively treated 57 patients with sunitinib 50 mg daily on a 4-week-on, 2-week-off schedule (41). Patient eligibility was expanded to both non-clear cell histology as well as clear cell histology with >20% sarcomatoid features. The objective response rate was a dismal 5% and progression free survival was 2.7 months; however, a subgroup analysis of the various tumor histologies revealed more promising results in the chromophobe RCC cohort, with a response rate of 40% and progression free survival of 12.7 months. The indolent nature of chromophobe tumors and favorable prognosis associated with them has been previously described (42).

Sunitinib has also demonstrated a moderate response in patients with translocation associated RCC. In a retrospective analysis of metastatic translocation associated RCC, the outcomes of 21 patients were evaluated by systemic treatment given (43). Patients treated with first line sunitinib (*n* = 11) had a significantly longer progression free survival than those treated with cytokine therapy (*n* = 9) (8.2 vs 2.0 months, respectively).

Another VEGF inhibitor, sorafenib, has been evaluated retrospectively for its utility in the setting of metastatic non-clear cell RCC. A recent study by Choueiri et al. analyzed 53 patients with papillary or chromophobe subtypes that were treated with either sunitinib or sorafenib (44). The response rate and median progression free survival of all patients were 10% and 8.6 months, respectively. Of note, patients with papillary tumors (*n* = 41) demonstrated a longer progression free survival when treated with sunitinib when compared to sorafenib (11.9 vs 5.1 months, respectively).

Bevacizumab is a monoclonal antibody that selectively inhibits the activity of human VEGF. The AVOREN trial (a phase 3 randomized controlled trial of bevacizumab plus interferon vs interferon alone) enrolled both patients with clear cell and mixed histology RCC (45). This trial showed a clear advantage of the bevacizumab arm, showing significantly longer PFS (10.2 vs 5.4 months) and higher ORR (31 vs 13%). Out of the 649 enrolled patients, 13% had some component of non-clear cell histology. A secondary analysis of this subgroup revealed a survival advantage of mixed histology patients with bevacizumab plus interferon vs bevacizumab alone (5.7 vs 2.9 months, respectively) (46).

### EGFR pathway

The deregulated activation of EGFR is thought to play a significant role in RCC tumorigenesis. The rationale for treatment of papillary RCC with EGFR inhibitors is based on data demonstrating cellular growth inhibition by blocking EGFR in the setting of wildtype VHL expression (47). Papillary renal tumors are molecularly defined by alterations in the *c-Met* oncogene (type 1) or the fumarate hydratase gene (type 2) and do not typically have the same VHL alterations as do their clear cell histology counterparts.

This prompted a phase 2 study conducted by Gordon et al. in which erlotinib was given as first line treatment to 45 patients with metastatic papillary RCC (48). Overall response rate was 11%, disease control rate (partial response and stable disease) was 64%, and median overall survival was 27 months. The investigators also subdivided this group by EGFR staining intensity on immunohistochemistry but there was no association with EGFR expression and time to progression or overall survival.

## Other Investigational Agents

### MET pathway

The *c-Met* oncogene encodes the receptor tyrosine kinase for hepatocyte growth factor (HGF). Once activated, this receptor promotes pathways involved in cell growth, survival, and invasion (49). An altered met oncogene (either due to activating mutations or gene amplification) is the defining molecular feature of type 1 papillary RCC (8, 50).

**Figure 3:**
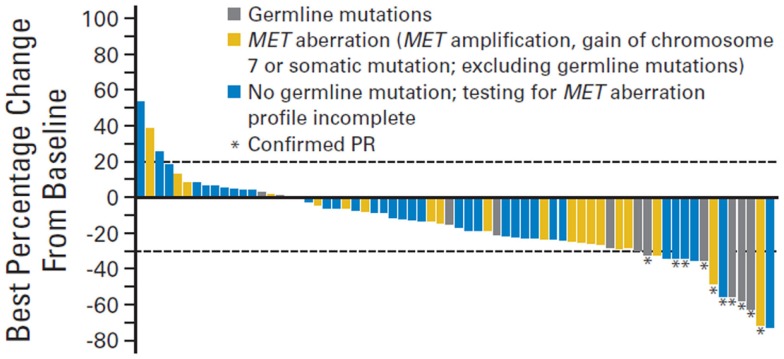
**Change in sum of longest tumor diameter**. PR, partial response.

This was the basis of a phase 2 trial conducted by Choueiri et al. that investigated the effect of foretinib (a dual MET/VEGFR inhibitor) in patients with metastatic papillary RCC (51). A total of 74 patients were enrolled in this trial. The overall response rate was 13% and median progression free survival was 9.3 months. Interestingly, patients with a germline or somatic *c-Met* alteration had a more drastic response to foretinib compared to those with wildtype *c-Met* (50 vs 9%, respectively) (Figure 3).

Inhibitors of *c-Met* have been studied in translocation associated RCC as well (52). Tivantinib (ARQ197), a selective inhibitor of *c-Met*, was recently investigated in a multicenter single arm phase 2 trial for treatment of microphthalmia transcription factor associated tumors (MiT’s). These tumors are characterized by altered expression of certain E-box binding transcription factors, leading to dysregulated growth of certain cell lines (53). The MiT family includes many tumors including translocation associated RCC, a malignancy of the kidney characterized by early age of onset and a high metastatic potential (54). Of the 47 patients enrolled in the tivantinib trial, 6 had translocation associated RCC. There were no partial responses and 3/6 (50%) patients had stable disease in the translocation associated RCC subgroup, and median progression free survival was 1.9 months.

### Proteasome pathway

Proteolysis is a critical component of normal cell growth and proliferation, and as such, when dysregulated can lead to unsuppressed cell cycle activity and tumor production (55). Normal proteolysis is carried out via the ubiquitin–proteasome system.

Recently bortezomib, an agent that inhibits the 26S proteasome, was studied in a phase 2 trial for treatment of advanced RCC. The trial included 37 patients with various tumor histologies (both clear cell and non-clear cell). An objective response rate of 11% was observed, but it is worth noting that the one patient with medullary RCC had an extraordinary response to therapy, showing a complete response after 7 months of treatment and remained without evidence of disease at 27 months (56). The investigators recommended further investigation of the role of ubiquitin–proteasome inhibitors in advanced medullary RCC.

### Chemotherapy options

The role of multi-agent chemotherapy for treatment of collecting duct RCC is actively being investigated due to its close biologic resemblance to upper tract urothelial carcinoma. Recently, a phase 2 trial of gemcitabine plus platinum salt for metastatic collecting duct carcinoma was conducted (57). The study evaluated the responses of 23 patients treated with this multi-agent chemotherapy regimen as first line therapy. Patients had an objective response rate of 26%, median progression free survival of 7.1 months, and median overall survival of 10.5 months. There are multiple case reports documenting the response of metastatic collecting duct and medullary RCC to multi-agent chemotherapy. Gollob et al. described a woman with advanced collecting duct carcinoma who had an 80% response to taxol/carboplatin therapy (58). Another patient with metastatic collecting duct carcinoma reported by Milowsky et al. had a 68% response to doxorubicin and gemcitabine (59). Lastly, three young patients with metastatic medullary RCC achieved a partial response to high dose methotrexate, vinblastine, doxorubicin, and cisplatin as reported by Rathmell et al. (60).

## Role of Surgery

There is a well-established clinical benefit of cytoreductive nephrectomy in the setting of metastatic RCC (61, 62). Although two landmark randomized trials studying the effect of cytoreductive nephrectomy were conducted prior to the widespread accepted use of targeted therapy, there still appears to be a survival benefit in patients with metastatic disease who are able to tolerate surgery. There are currently two ongoing trials addressing the clinical utility of cytoreductive nephrectomy in the era of targeted therapy: (1) the CARMENA study, which randomized patients into nephrectomy plus sunitinib vs sunitinib alone, and (2) the SURTIME study, which randomized patients into neoadjuvant sunitinib plus nephrectomy vs nephrectomy plus adjuvant sunitinib (63).

Kassouf et al. recently reviewed the M.D. Anderson Cancer Center experience of cytoreductive nephrectomies in non-clear cell disease (64). Of the 606 nephrectomies performed in patients with metastatic RCC, 92 had nccRCC. Patients with nccRCC were younger and had a higher incidence of nodal metastases. The disease specific survival was also worse in nccRCC patients undergoing cytoreductive nephrectomy when compared to ccRCC patients (9.7 vs 20.3 months). In contrast to these results, a review of the Mayo Clinic experience with cytoreductive nephrectomies (*n* = 505) reported by Carrasco et al. did not reveal an association of adverse survival in patients with non-clear cell disease (65).

Overall, the utilization of cytoreductive nephrectomy still plays an important role in metastatic RCC, regardless of histologic subtype. Aizer et al. recently investigated the role of cytoreductive nephrectomy in nccRCC through outcomes analysis of the SEER database (66). They found an overall RCC-specific and overall survival advantage among patients with nccRCC, who had undergone cytoreductive nephrectomy. When histologic subtypes were independently assessed, those with chromophobe and collecting duct pathology derived similar survival advantage to conventional clear cell tumors. While patients with papillary tumors had a decreased RCC-specific mortality after undergoing cytoreductive nephrectomy, the magnitude of this advantage was less than patients with ccRCC (HR 0.71 vs 0.48, respectively).

## Future Studies

Phase 3 trials for nccRCC are very difficult to conduct due to low disease prevalence. As such, clinical trials typically enroll both ccRCC and nccRCC patients, with stratification of both disease groups in each treatment arm. The following ongoing phase 3 trials are incorporating this strategy: NCT00326898 (adjuvant sunitinib vs adjuvant sorafenib after nephrectomy), NCT00492258 (adjuvant sorafenib vs placebo after nephrectomy), NCT01120249 (adjuvant everolimus vs placebo after nephrectomy) (63).

There are also a number of phase 2 studies focusing on nccRCC currently ongoing and/or recruiting participants. The therapeutic agents being investigated (either alone or in combination) include sunitinib (NCT01108445, NCT01185366, NCT00465179, NCT00979966, NCT01034878, NCT01219751, NCT01673386, NCT00326898, NCT01164228), sorafenib (NCT00326898), temsirolimus (NCT00979966), everolimus (NCT01108445, NCT01185366, NCT00830895, NCT01399918, NCT00688753, NCT01239342), Akt inhibitor MK2206 (NCT01239342), bevacizumab (NCT01399918), pazopanib (NCT01538238, NCT01767636), axitinib (NCT01798446), bortezomib (NCT00276614), tivozanib (NCT01297244, NCT01673386), crizotinib (NCT01524926), foretinib (NCT00726323), gemcitabine (NCT00491075, NCT00401128, NCT01164228), capecitabine (NCT01182142, NCT00226798), pemetrexed (NCT00491075), irinotecan (NCT00401128), fludarabine (NCT00027820, NCT00078858, NCT00243009), epothilone b (NCT00035243).

## Conclusion

There have been significant strides made in the treatment of metastatic RCC in the last decade, including the development of targeted therapies and the increased utilization of cytoreductive nephrectomy. While most prospective trials have included patients with all histologies of RCC, it is important to remember that the less common subtypes can display very different clinical behavior and response to therapy. Currently, there is no widely accepted first line therapy for metastatic nccRCC, and as such, enrollment in a clinical trial is the preferred option according to NCCN guidelines. Other considerations should be temsirolimus, sorafenib, sunitinib, pazopanib, axitinib, everolimus, bevacizumab, or erlotinib. Due to the low prevalence of this disease spectrum, it is of critical importance for there to be multi institutional collaboration in efforts to conduct prospective clinical trials of novel treatments for patients with metastatic nccRCC.

## Conflict of Interest Statement

The authors declare that the research was conducted in the absence of any commercial or financial relationships that could be construed as a potential conflict of interest. The Associate Editor Jose A. Karam declares that, despite having collaborated with author James J. Hsieh, the review process was handled objectively and no conflict of interest exists.
